# What psychological interventions are effective for the management of persistent physical symptoms (PPS)? A systematic review and meta‐analysis

**DOI:** 10.1111/bjhp.12613

**Published:** 2022-07-15

**Authors:** Katherine Swainston, Stacie Thursby, Blossom Bell, Hannah Poulter, Lorelle Dismore, Lee Copping

**Affiliations:** ^1^ Faculty of Medical Sciences School of Psychology Newcastle University Newcastle‐upon‐Tyne UK; ^2^ Northumbria Healthcare NHS Foundation Trust North Tyneside General Hospital North Shields UK; ^3^ School of Social Sciences, Humanities & Law Teesside University Middlesbrough UK

**Keywords:** anxiety, depression, meta‐analysis, persistent physical symptoms, psychological interventions, somatic symptoms, systematic review

## Abstract

**Objectives:**

Presentation of persistent physical symptoms is associated with increased health care utilization, yet clinical outcomes often remain suboptimal. This systematic review aimed to determine whether psychological interventions are effective for the management of PPS and if so, what are the features of the interventions and at what level of care are they delivered. The review also set out to establish which symptoms in those diagnosed with PPS can be effectively managed with psychological intervention.

**Methods:**

Studies were included if they clearly reported a psychological intervention, specified the study sample as adults with a diagnosis of persistent physical symptoms, included a comparator and as a minimum an outcome measure of somatic symptoms. Risk of bias was assessed using the EPHPP. Meta‐analysis was conducted to estimate the overall effect of interventions on somatic symptoms (the primary outcome), anxiety and depression (secondary outcomes).

**Results:**

Seventeen papers of varying quality indicated that psychological interventions can be effective for the management of somatic symptoms reported by individuals with PPS within a primary care setting. Psychological interventions were also found to be effective at reducing depression symptoms in individuals with PPS in twelve of the included studies. However, the meta‐analysis results suggest that the psychological interventions utilized within eleven of the included studies did not significantly impact anxiety symptoms.

**Conclusions:**

Psychological interventions have some success in managing somatic symptoms in PPS patients within primary care settings although their effects on other psychological symptoms is more mixed. The review highlights the importance of establishing a clearer diagnostic classification to inform treatment trajectories and the need for appropriate training and support within a multi‐disciplinary team to enable the provision of such therapies.

## BACKGROUND

*Persistent physical symptoms* (PPS) refers to persistent bodily complaints for which adequate examination does not reveal an organic pathology. Also referred to as *medically unexplained symptoms*, PPS accounts for at least 20% of GP consultations and 52% of referrals to secondary care (Jadhakhan et al., 2019). The extensive utilization of health services in this patient group costs the NHS £3.1 billion in direct fees, with total annual expenditure estimated to be £18 billion (Bermingham et al., 2010). At present, PPS is diagnosed based on two major clinical classification systems. The International Classification of Disease (ICD) which is inclusive of somatic and mental disorders, with PPS‐related syndromes such as irritable bowel syndrome and fibromyalgia placed in organ‐specific chapters; and the Diagnostic and Statistical Manual for Mental Health (DSM) which confines diagnoses to the domain of psychiatry (Burton et al., 2020). The uncertainty surrounding the diagnostic criteria of PPS often leads to unnecessary and unproductive investigations, contributing to the high healthcare costs.

Effective interventions are required for this patient group and the assessment of symptoms is imperative to informing their treatment trajectory. Due to the somatic nature of their complaints, patients will often attribute their symptoms to biomedical causes. However, it is important that aetiological relevant psychological factors such as depression, anxiety, traumatic experiences and stressful life events are also assessed (Häuser et al., 2013; Jones et al., 2009; Reuber et al., 2005). A subset of PPS patients find it difficult to consider psychological therapy as an appropriate treatment (Kleinstäuber et al., 2017), which facilitates conflict‐laden relations and communication breakdown between physician and patient (Murray et al., 2016). Communication breakdown can be characterized by patients appearing demanding or premeditated to seek answers in conjunction with their fixed health beliefs and assumptions, particularly seeking somatic causes for their complaints (Röhricht et al., 2019; Wileman et al., 2002). This consequently imposes pressure on physicians to prescribe pharmacological therapies, especially when patients vehemently complain about their symptoms (Salmon et al., 2007).

A lack of health care providers with a specialist interest in PPS and pressure on GP consultation time can lead to reports of dissatisfaction by both physicians and patients. NICE (2021) best practice guidance recommends holistic, multi‐disciplinary care involving medical, social and psychological provision for the management of PPS‐related syndromes and symptoms. Yet, fragmented organizational structures will often leave patients being repeatedly referred for investigation and consultation (Burton et al., 2012), finding that care can be suboptimal and pathways ill‐defined and complex. Burton et al. (2020) propose a new classification for PPS, terming it Functional Somatic Disorders (FSD), asserting that it should not favour somatic disease or mental disorder. Instead, the development and maintenance of FSD symptoms will be attributed to the interaction between various processes and not to a singular aetiological mechanism.

The extra level of depth offered by the FSD classification is acknowledged through its incorporation of co‐existing psychological symptoms and somatic illness (Burton et al., 2020). This is important for clinicians, to ensure that diagnoses encapsulate all specialist areas and to engage patients who may be hesitant to accept psychological therapy from clinicians who appear ‘too psychological’ in the initial stages of diagnosis. Burton et al. (2020) enunciate that dysfunctional psychological (cognitive, affective) and behavioural features may accurately characterize disorders or direct treatment. This is reflected in treatments modelled on psychological therapies for the management of PPS, whereby interventions such as psychodynamic therapy and cognitive‐behavioural therapies have been utilized to address psychological presentations including health anxiety, catastrophizing, somatization amplification and negative affect (Mobini, 2015). Moreover, predisposing biological vulnerabilities including immune suppression, hypothalamic–pituitary–adrenal axis and autonomic dysregulation can be aggravated by psychosocial stressors, for example adverse childhood experiences, interpersonal conflicts (Johnson, 2008).

Recognizing that these processes may be shared across individual syndromes yet differ in presentation (Bourke et al., 2015) is an essential part of the consultation. A shared understanding of these processes will enable congruence between patients and physicians and their views towards illness, thereby diminishing challenges that current conflicting classifications perpetuate (McAndrew et al., 2018). Outcomes will therefore be different between groups whether they received their diagnosis and/or the management of their PPS is in primary or secondary care. The implication of this is that we should diagnose earlier and minimize medicalization, utilizing psychological therapies where possible. Despite the use of some psychological interventions shown to facilitate the management of PPS, there is limited research, and studies that do exist often contain small sample sizes. A systematic review is required to critically appraise existing evidence and assess the efficacy of psychological therapies that have been used for the management of PPS.

### The present study

This review aims to establish (a) whether psychological intervention is effective for the management of PPS and if so, are any specific interventions more effective than others; (b) at what level of care are psychological interventions for PPS most effective; (c) which symptoms in those diagnosed with PPS can be effectively managed with psychological intervention? This review addresses these questions and provides guidance for those developing services to manage PPS.

## METHODS

The review was pre‐registered on PROSPERO (CRD42021237394).

### Search strategy and selection criteria

Searches were performed on PsycINFO, MEDLINE, Scopus, CINAHL, EMBASE, Psychology and Behavioural Science Collection, The Allied and Complementary MEDicine, Proquest Nursing and Allied Health Source. The searches were supplemented by the following grey literature sources – MEDNAR and Google Scholar. Searches were undertaken in March 2021. There were no date restrictions and we included papers published in any language provided they had an English title and abstract and could be translated. Search filters used were for condition (persistent physical symptoms, medically unexplained symptoms), intervention (psychological, psychosocial, psychology, therapy) and study type (intervention, trial, experiment) (Table A1).

Studies were included if they (a) clearly reported a psychological intervention, (b) included adult human participants, (c) specified the study sample as individuals with a diagnosis of persistent physical symptoms/medically unexplained symptoms, (d) included a comparator (a control group, an alternative intervention, measurement over time), (e) in any setting, (f) were any study design (randomized controlled trials, non‐randomized controlled trials, pre‐post studies; natural experiments), (g) included an outcome measure of somatic symptoms.

### Screening

Two authors screened each reference using Rayyan software. At title/abstract screening any reference recorded as ‘include’ or ‘maybe’ by at least one screener was reviewed at the full text stage. Full texts were screened by two additional authors and any disagreements were resolved in discussion with a third screener.

### Data extraction

Data were extracted by two authors using a data extraction tool recording: details of the sample (e.g. age, gender), the psychological intervention used (e.g. CBT, ACT), level of care at which the intervention was delivered (e.g. primary care, secondary care), outcome measures assessed (somatic symptoms and any additional outcomes e.g. depression), study details (e.g. design, quality assessment), study results/findings (including effect sizes).

Study quality was assessed using the EPHPP quality assessment tool for quantitative studies (Thomas et al., 2004). Two authors independently conducted quality appraisal and any disagreements were resolved by a third author. The extracted study findings were checked by an additional data extractor.

### Meta‐analysis

Quantitative studies that contained either a pre/test post‐test intervention group or an intervention versus control were considered for inclusion in a meta‐analysis to estimate the overall effect of interventions on somatic symptoms (the primary outcome), anxiety and depression (secondary outcomes).

Studies meeting the criteria were identified and relevant data extracted in the form of group *n*, mean and standard deviation. Some studies did not include standard deviations, but these were calculable from provided standard errors or confidence intervals. Studies also utilized different metrics and so a standardized mean difference was used in analysis (Hedges G). As different outcome measures vary in whether a reduction in symptoms is represented by a higher or lower score, all effect sizes were presented so that a lower score (a negative effect size) represented a reduction in somatic symptoms.

All analyses were conducted using a random effects model (allowing variation to be attributable to influences beyond sampling differences) implementing the more robust Hartung‐Knapp method of estimation (Hartung & Knapp, 2001).

Q statistics were implemented as an indicator of heterogeneity (Hedges & Olkin, 1985). These follow a chi‐square distribution of *k*−1 degrees of freedom and are calculated via the following method:

$$Q=\sum_{i=1}^{k}w\left(d_{i}-\bar{d}\right)\begin{array}{c} 2 \end{array}$$

where *k* represents the number of effect sizes in the analysis and *w* = 1/*v* (*v* = (*N*_1_ + *N*_2_)/*N*_total_ + *d* ^2^/2(N_total_)).

Significant values for *Q* are only indicative of heterogeneity. *I* ^2^ was also used alongside of it to give a sense of proportionality to inconsistencies across effect sizes (Higgins et al., 2003). Where significant heterogeneity was identified, moderator analysis was conducted to attempt to identify pertinent sources of it. This provides an indicator of what potential study factors may be affecting variation in effect sizes across studies in the analysis. In this study, several moderators that were measurable across all studies were employed. These included: year of publication, study quality rating, type of measure employed, CBT versus Non‐CBT intervention, control sample versus non‐control sample and country the study was conducted in. *Q*_*b*_ was used to test if G differed between subgroups (a test analogous to an *F* statistic).

Publication bias was examined for the primary outcomes using a funnel plot.

All analyses were conducted in *R* (V3.6.0) using the *metafor* package.

## RESULTS

**FIGURE B1:**
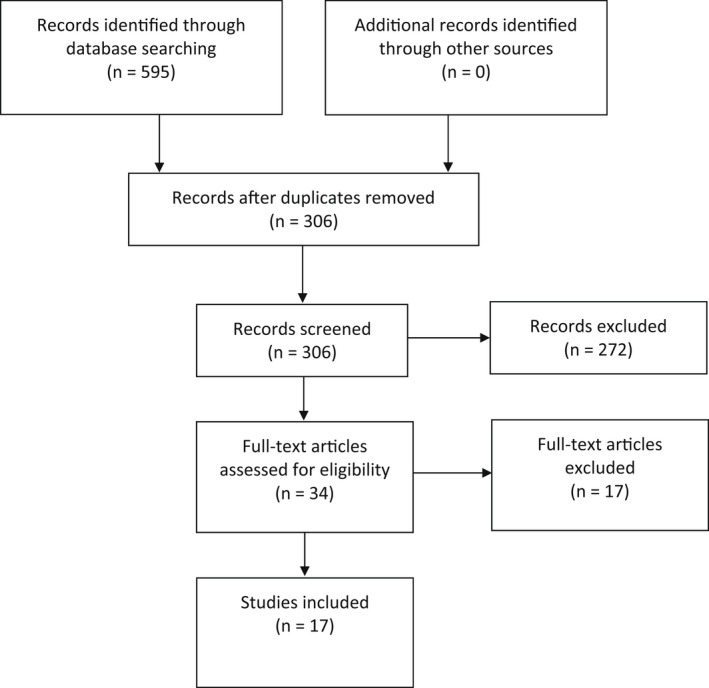
Flow diagram of included/excluded studies

Figure B1 depicts the results of the search and screening processes. The search process identified 595 papers. After the removal of duplicate papers, 306 titles and abstracts were screened. Screening was conducted by all authors. Studies that did not meet the inclusion criteria were excluded (*N* = 272). The main reasons for exclusion at this stage were that no psychological intervention had occurred, and the study sample did not include individuals diagnosed with PPS/MUS. Full texts were assessed for eligibility (*N* = 34) leaving 17 articles for inclusion in the review (Tables A1 and C1).

### Study characteristics

The 17 papers that met the inclusion criteria reported on 829 participants experiencing PPS who received psychological intervention. Fifteen studies were randomized controlled trials (Aiarzaguena et al., 2007; Escobar et al., 2007; Gili et al., 2014; Katsamanis et al., 2011; Martin et al., 2007; Menon et al., 2017; Röhricht et al., 2019; Sattel et al., 2012; Schröder et al., 2012; Schröder et al., 2013; Sitnikova et al., 2019; Sumathipala et al., 2000; Sumathipala et al., 2008; Van Ravesteijn et al., 2013; Wortman et al., 2016). Control participants in these studies reported PPS but did not receive psychological intervention. Two studies were pre/post designs reporting a treatment arm only (Hubley et al., 2017; Kleinstäuber et al., 2017).

Studies were based in Denmark (Schröder et al., 2012), Germany (Kleinstäuber et al., 2017; Martin et al., 2007; Röhricht et al., 2019; Sattel et al., 2012; Schröder et al., 2013), the Netherlands (Sitnikova et al., 2019; Van Ravesteijn et al., 2013; Wortman et al., 2016), Spain (Aiarzaguena et al., 2007; Gili et al., 2014), India (Menon et al., 2017), Sri Lanka (Sumathipala et al., 2000; Sumathipala et al., 2008) and the USA (Escobar et al., 2007; Hubley et al., 2017; Katsamanis et al., 2011).

Psychological interventions included cognitive behavioural therapy (CBT) or cognitive behavioural therapy‐based interventions (*N* = 11; Escobar et al., 2007; Gili et al., 2014; Kleinstäuber et al., 2017; Martin et al., 2007; Menon et al., 2017; Schröder et al., 2012; Schröder et al., 2013; Sitnikova et al., 2019; Sumathipala et al., 2000; Sumathipala et al., 2008; Van Ravesteijn et al., 2013). Van Ravesteijn and colleagues reported utilizing mindfulness‐based CBT specifically while Martin et al. (2007) and Menon et al. (2017) referred to the use of single session CBT. The remaining studies utilized psychodynamic therapies including brief psychodynamic interpersonal therapy (Sattel et al., 2012) and multi‐modal psychosomatic therapy (Wortman et al., 2016); group body psychotherapy (BPT; Röhricht et al., 2019), a psychophysiological intervention (Katsamanis et al., 2011), behavioural consultation (Hubley et al., 2017) and a psychosocial communication intervention (Aiarzaguena et al., 2007).

All studies were reported to be conducted within a primary care setting, either via a GP practice or outpatient clinic.

### Risk of bias

Five studies were classified as strong quality, ten studies as moderate and two studies were rated as weak.

### Main results

In accordance with the review inclusion criteria, the seventeen included studies reported on the effectiveness of a psychological intervention on somatic symptoms. Meta‐analysis has been conducted on this as a primary outcome measure. In addition to somatic symptoms, eleven studies examined the impact of psychological interventions on anxiety symptoms, and twelve studies included an outcome measure of depression symptoms. The results of the meta‐analysis for these secondary outcomes are provided. A minority of studies reported additional outcome measures (e.g. pain, hyperventilation, number of hospital visits), however the small number of studies (typically *N* = 1) and variation in measures rendered these outcomes unsuitable for meta‐analysis.

### Somatic symptoms

**FIGURE 1:**
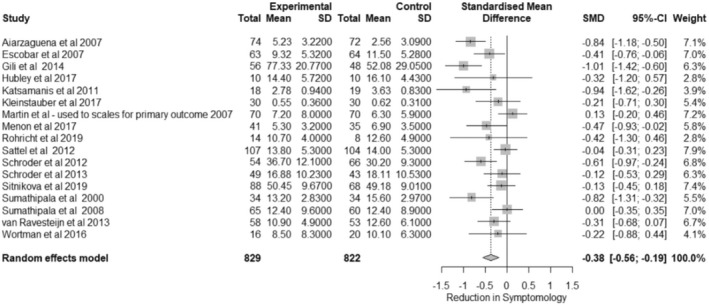
Forest plot of somatic symptoms data. *Note:* In Hubley et al. (2017) and Kleinstäuber et al. (2017) the pre test mean is in the control column and the post‐test in the experimental

Studies examining the effects of an intervention on somatic symptoms were aggregated across 17 studies. There were several measures employed by studies to measure somatic symptoms (for specific details on how these record somatic symptoms, readers are encouraged to examine these clinical scales themselves). These included the Four‐Dimensional Symptom Questionnaire (4DSQ), the Brief Symptoms Inventory (BSI), the Clinical Global Impression Scale for Somatization Disorder (CGI‐SD), the Patient Health Questionnaire (PHQ‐15), RAND‐36, Short Form – 26 (SF‐36) and the Screening of Somatoform Symptoms 7 (SOMS‐7). Figure 1 shows the forest plot for the effect of interventions on somatic symptoms. Means and standard deviations are presented as raw scores whilst the effect size is presented as the standardized mean difference (SMD) Hedges G (alongside its 95% confidence interval).

The overall effect size was calculated as *G* = −0.38 [−0.56; −0.19], *p* < .001 suggesting that interventions do have a small, statistically significant impact on reducing somatic symptoms.

There was a significant amount of heterogeneity in this analysis:

*Q*(16) = 46.80, *p* < .001, *I* ^*2*^ = 65.8% [42.9%; 79.5%]. Moderator analysis was conducted to examine potential underlying sources of this heterogeneity. Table 1 illustrates the effects of these moderators.

**TABLE 1:** Moderation analysis of somatic symptoms

Moderator	Group	*k*	SMD [95%CI]	*Q*	*I* ^2^
Quality	Strong (1)	5	−0.31 [−0.71; 0.10]	11.06	63.8%
	Moderate (2)	10	−0.46 [−0.74; −0.18]	30.15	70.1%
	Weak (3)	2	−0.15 [−0.56; 0.25]	0.05	0.0%
	Between groups	*Q*(2) = 6.58, *p* < .05			
Year	2000	1	−0.82 [−1.31; −0.32]	0.00	0.0%
	2007	3	−0.37 [−1.58; 0.83]	16.12	87.6%
	2008	1	0.00 [−0.35; 0.35]	0.00	0.0%
	2011	1	−0.94 [−1.62; −0.26]	0.00	0.0%
	2012	2	−0.31 [−3.92; 3.30]	5.97	83.2%
	2013	2	−0.22 [−1.42; 0.98]	0.45	0.0%
	2014	1	−1.01 [−1.42; −0.60]	0.00	0.0%
	2016	1	−0.22 [−0.88; 0.44]	0.00	0.0%
	2017	3	−0.35 [−0.73; 0.03]	0.60	0.0%
	2019	2	−0.17 [−1.33; 0.99]	0.36	0.0%
	Between groups	*Q*(9) = 24.98, *p* < .01			
Measure	4DSQ	1	−0.22 [−0.88; 0.44]	0.00	0.0%
	BSI	2	−0.39 [−5.58; 4.80]	6.96	85.6%
	CGI‐SD	1	−0.94 [−1.62; −0.26]	0.00	0.0%
	PHQ‐15	6	−0.25 [−0.46; −0.05]	4.34	0.0%
	RAND‐36	1	−0.13 [−0.45; 0.18]	0.00	0.0%
	SF‐36	3	−0.81 [−1.29; −0.33]	2.08	3.8%
	SOMS‐7	3	−0.02 [−0.45; 0.42]	1.49	0.0%
	Between groups	*Q*(6) = 33.43, *p* < .001			
Intervention	CBT	11	−0.34 [−0.58; −0.11]	30.24	66.9%
	Non‐CBT	6	−0.46 [−0.58; −0.11]	16.14	68.5%
	Between groups	*Q*(1) = .37, *p =* .544			
Design	Control	15	−0.39 [−0.59; −0.19]	46.55	69.9%
	No control	2	−0.23 [−0.85; 0.38]	0.05	0.0%
	Between groups	*Q*(1) = 2.12, *p =* .145			
Country	Denmark	1	−0.61 [−0.97; −0.24]	0.00	0.0%
	Germany	5	−0.04 [−0.22; 0.14]	2.25	0.0%
	India	1	−0.47 [−0.93; −0.02]	0.00	0.0%
	Netherlands	3	−0.21 [−0.45; 0.04]	0.47	0.0%
	Spain	2	−0.91 [−1.93; 0.12]	0.37	0.0%
	Sri Lanka	2	−0.39 [−5.58; 4.80]	6.96	85.6%
	U.S.	3	−0.50 [−1.15; 0.15]	2.01	0.5%
	Between groups	*Q*(6) = 79.40, *p <* .001			

Abbreviations: 4DSQ, (Four‐Dimensional Symptom Questionnaire); BSI, Brief Symptoms Inventory; CGI‐SD, Clinical Global Impression Scale for Somatization Disorder; PHQ‐15, Patient Health Questionnaire; RAND‐36, SF‐36 (Short Form ‐ 26); SOMS‐7, Screening of Somatoform Symptoms 7.

As illustrated in Table 1, several variables appear to account for significant proportions of heterogeneity in the outcomes. These include study quality, year, measurement instrument and country (*p* < .05 in all cases).

### Secondary outcomes

#### Anxiety

**FIGURE 2:**
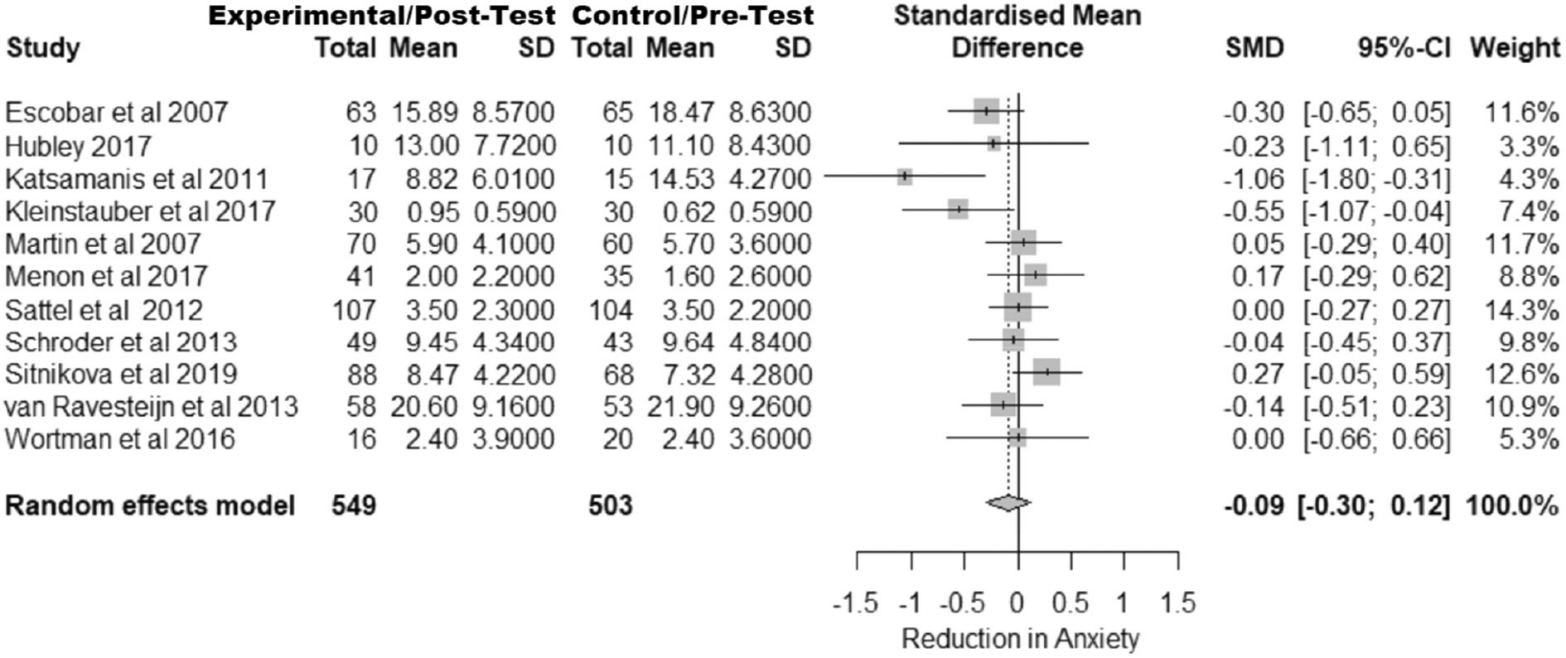
Forest plot of anxiety outcomes. *Note:* In Hubley et al. (2017) and Kleinstäuber et al. (2017) the pre test mean is in the control column and the post‐test in the experimental

Eleven studies examined the impact of interventions on anxiety symptoms. As previously, the standardized mean Hedges *G* was used as the unit for meta‐analysis. Figure 2 shows the forest plot for the effect of interventions on anxiety symptoms.

The overall effect size was calculated as *G* = −0.09 [−0.30; 0.12], *p* = .343 suggesting that these interventions do not significantly impact anxiety symptoms.

Heterogeneity in this analysis was also non‐significant, *Q*(10) = 18.14, *p* = .053, *I* ^*2*^ = 44.9% [0.0%; 72.7%] and so moderator analysis was not conducted.

#### Depression

**FIGURE 3:**
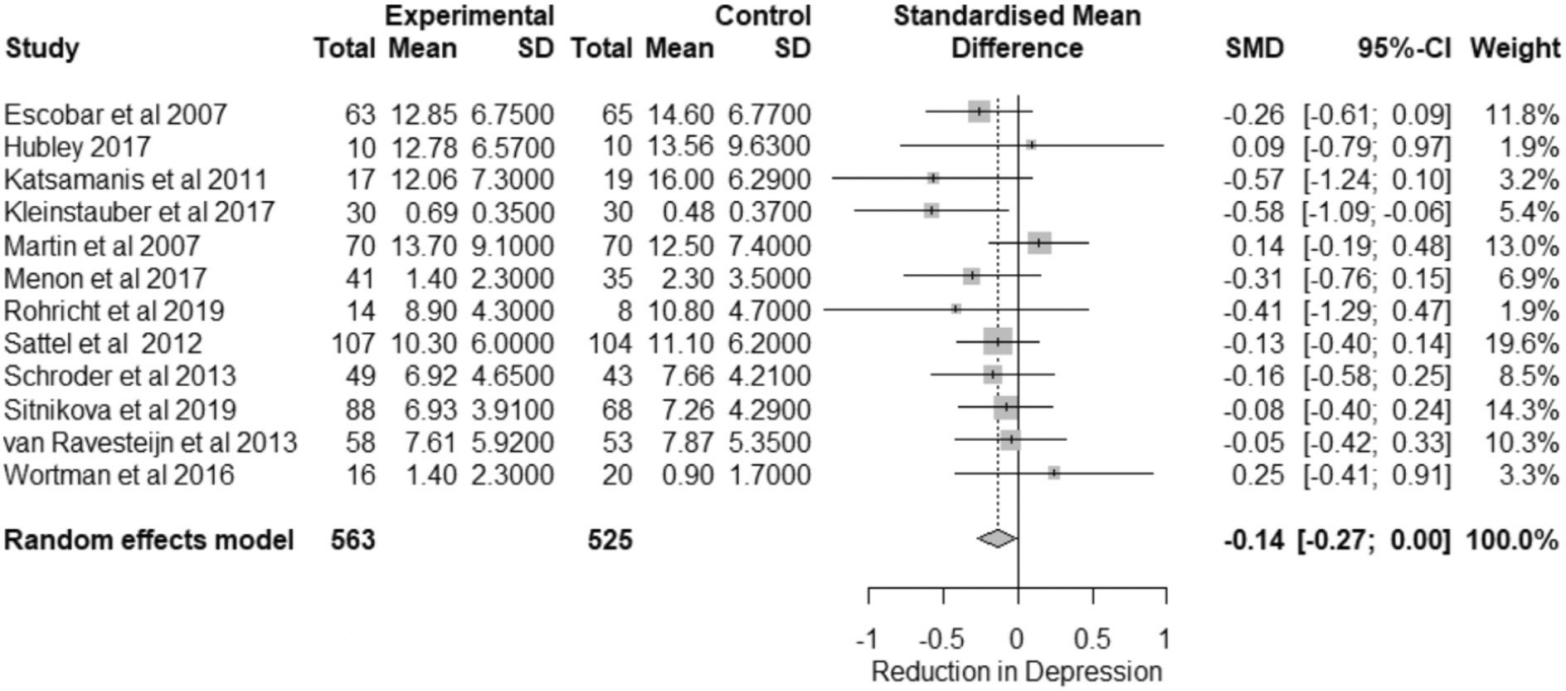
Forest plot of depression outcomes. *Note:* In Hubley et al. (2017) and Kleinstäuber et al. (2017), the pre test mean is in the control column and the post‐test in the experimental

Twelve studies also examined the impact of interventions on depression symptoms. The same procedure as in previous analyses was used to produce a forest plot (Figure 3) showing the effect of interventions on depression symptoms.

The overall effect size was calculated as *G* = −0.14 [−0.27; −0.00], *p* < .05 suggesting that interventions do have a small, statistically significant impact on reducing the impact of depression symptoms in participants.

There was no significant heterogeneity in this analysis, *Q*(11) = 10.40, *p =* .494, *I* ^*2*^ = 0.0% [0.0%; 55.9%]. Moderator analysis was thus not conducted.

#### Publication bias

**FIGURE 4:**
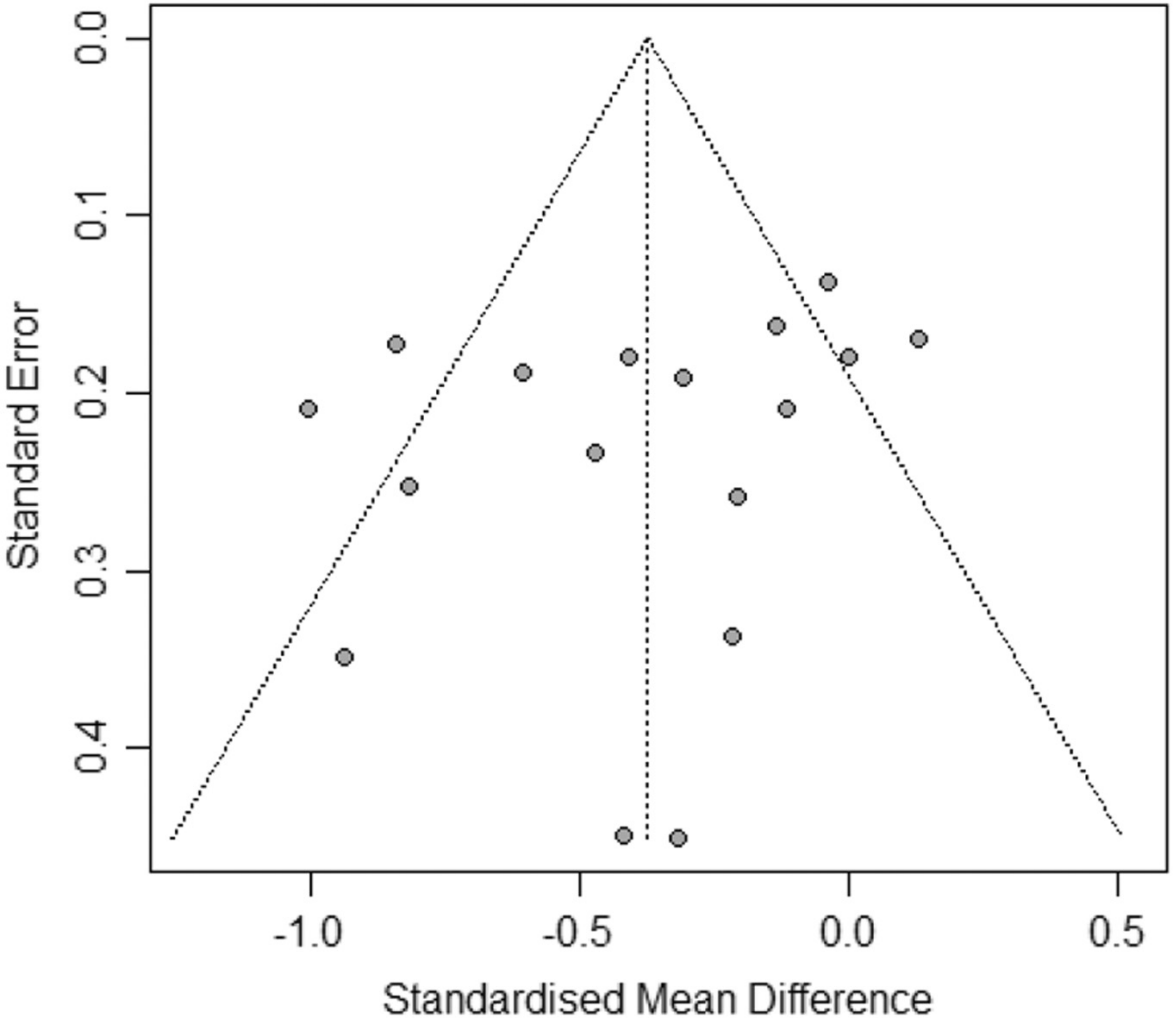
Funnel plot of all studies

Publication Bias was examined using a funnel plot. Figure 4 shows the results of this and suggests that publication bias is not a major issue in this study.

## DISCUSSION

This systematic review identified 17 studies reporting on the effectiveness of psychological interventions delivered within a primary care setting to patients with Persistent Physical Symptoms. The results indicate that psychological interventions were effective in reducing somatic and depression symptoms but there was no statistically significant effect on anxiety symptoms. CBT, psychodynamic and communication‐based interventions were reported to be effective though moderation analysis identified that there was no significant difference on somatic outcomes between a CBT or non‐CBT based intervention.

Patients with PPS report more social isolation, psychological distress, more functional and occupational impairment, poorer quality of life and overall impaired health status in comparison to general practice patients not meeting the criteria for PPS (Creed et al., 2012; Dirkzwager & Verhaak, 2007; Woo, 2010). Depression and anxiety are common in patients with PPS (Burton et al., 2011) and are indicators of improvement in recovery (Geraghty & Scott, 2020). Accordingly, developing better management strategies for patients with PPS is a health care priority.

Improving the treatment of patients with PPS has the potential to provide better, more appropriate and more cost‐effective care (Burton et al., 2011). Many patients with PPS receive unnecessary somatic investigations and treatment in primary care (Ring et al., 2004). Physical interventions are more often proposed by GPs than patients, with this trend attributed to patients pressuring doctors for somatic management (Ring et al., 2005). Psychological factors are oftentimes therefore omitted or completely ignored (Orzechowska et al., 2021). Avoiding unnecessary symptomatic intervention is warranted and GPs and primary care providers need interventions that reflect the ways patients influence their decisions and that are based on a clear understanding of the goals that shape patients' presentations and doctors' responses (Ring et al., 2004).

Our data support the use of psychological interventions delivered in primary care as an approach to reduce somatic and depression symptoms rather than a dualistic approach for treatment of PPS. Many of the included studies adopted a CBT approach and Deary et al. (2007) have developed a CBT model of MUS, whereby the patient is challenged on the origins of symptoms such as pain or fatigue and perpetuates their own illness by holding on to beliefs in an ‘organic’ illness. While the data showed no heterogeneity between type of intervention (CBT and non‐CBT) the results are promising for the development of psychological interventions incorporating a cognitive behavioural component.

Intervention delivery in the included studies was typically by either a trained psychologist or therapist or the patients GP. GPs in the United Kingdom must be capable of assessing patients with PPS and accordingly establishing a well‐defined clinical diagnosis of PPS would aid identification of patients who may benefit from a systematic, qualified approach to the complex problems they experience (Rosendal et al., 2005). The lack of guidelines for treatment and management of this group (Czachowski et al., 2011) further adds to the challenges faced but appropriate training and supervision could address GP's lack of confidence and improve their knowledge of PPS (Scope et al., 2021). The relationship between patients and service providers is key to the success of interventions, whilst poor communication between parties, and beliefs and attitudes held by both can produce barriers (Scope et al., 2021). Incorporating psychological expertise in the referral pathways to support the delivery of psychosocial interventions within primary care settings could break down communication barriers and facilitate the effective management of PPS.

### Limitations of the review

A number of limitations of this review must be acknowledged. Firstly, while the funnel plot examining publication bias in primary outcomes suggests this may not be an issue in this area of treatment, it should be noted that the number of studies included for analysis was small (*k* < 20) and so this examination is somewhat unreliable (Borenstein et al., 2009). Secondly, the included studies used a range of measures for somatic symptoms, anxiety and depression. Our moderation analysis indicates that studies which used the BSI were more likely to show stronger outcomes for PPS interventions on somatic symptoms. The studies utilizing the BSI were undertaken in Sri Lanka and accordingly differences may be attributable to variation in healthcare systems. Van Driel et al. (2018) studied somatization questionnaires for PPS in older adults and found that the BSI is superior for this population for interventions delivered within a primary care setting. The efficacy of other measures may therefore be questionable.

One indicator which was not assessed in detail was severity of PPS symptoms as a moderator of intervention effect. Within the UK IAPT evidence‐based pathways exist in primary care for individuals with mild‐ to moderate PPS symptoms, yet critics argue that individuals with more complex symptoms may not be served by these pathways (Chew‐Graham et al., 2017). Indeed, patients presenting with PPS in primary care represent a broader spectrum of severity than patients seen in a specialist setting (Rosendal et al., 2005).

The length of intervention was also not assessed as part of this review as variation in protocol was too large for effective moderation analysis. While some studies opted for a single‐session therapy approach, others had 6 or 8 sessions of psychological intervention either weekly or over the course of several months.

### Areas for future research

The data did not support the use of psychological interventions for reductions in anxiety, and therefore interventions reducing the anxiety reported by PPS patients are yet to be determined. If patients are to be supported by the use of psychosocial interventions led by GPs and psychologists, a classification of symptoms that better supports clinical decision‐making is needed; this may prevent patients who may be missed. Rosendal et al. (2017) have proposed a new approach to classification of symptoms in primary care that better supports clinical decision‐making, creates clearer communication and provides scientific underpinning of research to ensure effective interventions (Rosendal et al., 2017). However, this approach requires investigations within primary care populations. Further investigation of the most effective intervention duration is needed.

## CONCLUSION

PPS are common in general practice and are part of a GP's work. With appropriate training and support, interventions can be carried out by GPs in the primary care setting, which, while may not work for all symptoms (i.e. anxiety), may provide other symptom relief for patients. GPs may benefit from a multi‐disciplinary team approach to help manage PPS patients although further work on how this approach could be implemented is perhaps first required.

## AUTHOR CONTRIBUTIONS

**Katherine Swainston:** Conceptualization; formal analysis; investigation; methodology; project administration; supervision; validation; writing – original draft; writing – review and editing. **Stacie Thursby:** Investigation; methodology; validation; writing – original draft; writing – review and editing. **Blossom Bell:** Data curation; investigation; methodology; writing – review and editing. **Hannah Poulter:** Investigation; methodology; writing – review and editing. **Lorelle Dismore:** Conceptualization; investigation; methodology; validation; writing – original draft; writing – review and editing. **Lee Copping:** Formal analysis; investigation; methodology; validation; writing – original draft; writing – review and editing.

## CONFLICT OF INTEREST

None.

## Data Availability

The data that support the findings of this review are available from the corresponding author, KS, upon reasonable request.

## APPENDIX A

**TABLE A1:** Search strategy

Host	Database	Search no.	Search term	Results
EBSCO	MEDLINE, psychINFO, AMED, P&BSC, CINAHL	S1	AB ‘persistent pain symptoms’ OR AB ‘medically unexplained symptoms,	
		S2	Intervention OR trial OR experiment	
		S3	Psychological OR Psychosocial OR Psychology OR Therapy	
		S4	S1 AND S2 AND S3	
		S5	S4 with limiters: English & Journal	
		S6	Automatic removal of duplicates	
ProQuest	Nursing & Allied Health Source	S1	ab(‘persistent pain symptoms’) OR ab(’medically unexplained symptoms“)	
		S2	ab(intervention) OR ab(trial) OR ab(experiment)	
		S3	Ab(psychological) OR ab(psychosocial) OR ab(psychology) OR ab(therapy)	
		S4	S1 AND S2 AND S3	
	Scopus	S1	(TITLE‐ABS‐KEY(“medically unexplained symptoms”) OR TITLE‐ABS‐KEY(“persistent pain symptoms”))	
		S2	(TITLE‐ABS‐KEY(intervention) OR TITLE‐ABS‐KEY(trial) OR TITLE‐ABS‐KEY(experiment))	
		S3	(TITLE‐ABS‐KEY(psychological) OR TITLE‐ABS‐KEY(psychosocial) OR TITLE‐ABS‐KEY (psychology) OR TITLE‐ABS‐KEY (therapy))	
		S4	S1 AND S2 AND S3 with limiters: English & Journal	
Ovid	EMBASE	S1	(“persistent pain symptoms” or “medically unexplained symptoms”).ab.	
		S2	(intervention or trial or experiment).ab.	
		S3	(psychological or psychosocial or psychology or therapy).ab.	
		S4	S1 AND S2 AND S3	
		S5	S4 with limiters: Article	

## APPENDIX C

**TABLE C1:** Table of included studies

	Paper	Country	Quality score	Intervention *(features)*	Somatic measure	Anxiety	Depression	Other outcome(s)
1	Aiarzaguena et al. (2007)	Spain	2	Non‐CBT Psychosocial communication intervention	SF‐36			
				*(6 × 30 min consultation over a 5 month period)*				
2	Escobar et al. (2007)	USA	1	CBT	PHQ‐15	HAM‐A	HAM‐D	Pain (VAS)
				*(10 sessions over 3 months; each session lasting 45–60 min)*				
3	Gili et al. (2014)	Spain	2	CBT	SF‐36			
				*(10 × 90 min sessions over 10 weeks)*				
4	Hubley et al. (2017)	USA	2	Non‐CBT	PHQ‐15	GAD‐7	PHQ‐9	
				Behavioural consultation intervention				
				*(3 sessions)*				
5	Katsamanis et al. (2011)	USA	2	Non‐CBT	CGI‐SD	HAM‐A	HAM‐D	General health
				Psychophysiological intervention				
				*(10 sessions)*				
6	Kleinstäuber et al. (2017)	Germany	3	CBT *(25 × 50 min sessions)*	SOMS‐7 T	BSI	BDI‐II	
7	Martin et al. (2007)	Germany	2	CBT (Single session)	BSI	WI	BDI	Health‐related internal control
				*(3–4 h of intervention)*				Sick leave days
8	Menon et al. (2017)	India	1	CBT (with guided muscular relaxation; Single session)	PHQ‐15	GAD	PHQ‐9	Number of hospital visits and number of work days lost (but full data not present)
				*(1 × 30 min)*				
9	Röhricht et al. (2019)	Germany	2	Non‐CBT	PHQ‐15		PHQ‐9	
				Group Body Psychotherapy (BPT)				
				*(1 × 90 min session over 20 weeks)*				
10	Sattel et al. (2012)	Germany	1	Non‐CBT	PHQ‐15	WI‐7	PHQ‐9	
				Brief psychodynamic interpersonal psychotherapy				
				*(12 × approx. 45 min sessions)*				
11	Schröder et al. (2012)	Denmark	2	CBT	SF‐36			
				*(9 × 3.5 h sessions over 4 months)*				
12	Schröder et al. (2013)	Germany	2	CBT	SOMS	HADS‐A	HADS‐D	Physical functioning and mental health
				*(8 × 90 min sessions, weekly)*				
13	Sitnikova et al. (2019)	Netherlands	3	CBT	RAND‐36	HADS‐A	HADS‐D	
				*(6 × 30 min sessions)*				
14	Sumathipala et al. (2000)	Sri Lanka	1	CBT	BSI			
				*(6 × 30 min sessions over 3 months)*				
15	Sumathipala et al. (2008)	Sri Lanka	1	CBT	BSI			Number of complaints and patient‐initiated consultations
				*(6 × 30 min sessions over 9 weeks)*				
16	Van Ravesteijn et al. (2013)	Netherlands	2	CBT (mindfulness based)	PHQ‐15	WI	PHQ‐9	
				*(8 × 2.5 h weekly sessions of MBCT plus 6 days a week practice for 45 min)*				
17	Wortman et al. (2016)	Netherlands	2	Non‐CBT	4DSQ	4DSQ	4DSQ	Distress, hyperventilation, level of general functioning
				Multimodal psychosomatic therapy				
				(9 *×* 45 min session)				
